# Comparative Analyses of the Gut Microbiota in Growing Ragdoll Cats and Felinae Cats

**DOI:** 10.3390/ani12182467

**Published:** 2022-09-18

**Authors:** Zongjie Li, Di Di, Qing Sun, Xiaohui Yao, Jianchao Wei, Beibei Li, Ke Liu, Donghua Shao, Yafeng Qiu, Haixia Liu, Zhanjun Cheng, Zhiyong Ma

**Affiliations:** 1 Shanghai Veterinary Research Institute, Chinese Academy of Agricultural Science, Shanghai 200241, China; 2 Nanjing Policedog Insitute of the Ministry of Public Security, Nanjing 210012, China

**Keywords:** growing cat, Ragdoll cat, Felinae cat, gut microbiota, morphological portraits

## Abstract

**Simple Summary:**

Accumulating studies have revealed that the gut microbiota had intimate relations with the animal gastrointestinal tract diseases. Through regulating the development of the host’s intestinal immune system, the gut microbiota could directly influence the host’s intestinal function. In the current study, the gut microbiota of Ragdoll cats and Felinae cats were investigated and compared. Results demonstrated the diversity and richness of the gut microbiota in the Felinae cats were much higher than in the Ragdoll cats. However, the relative abundances of beneficial microbes in the Ragdoll cats were much higher than those in the Felinae cats. In all, different genetic portraits determined the different microbial communities in the feline gut. The candidate probiotics isolated in the growing cat’s gut might be applied to treat the gastrointestinal tract diseases.

**Abstract:**

Today, domestic cats are important human companion animals for their appearance and favorable personalities. During the history of their domestication, the morphological and genetic portraits of domestic cats changed significantly from their wild ancestors, and the gut microbial communities of different breeds of cats also apparently differ. In the current study, the gut microbiota of Ragdoll cats and Felinae cats were analyzed and compared. Our data indicated that the diversity and richness of the gut microbiota in the Felinae cats were much higher than in the Ragdoll cats. The taxonomic analyses revealed that the most predominant phyla of the feline gut microbiota were Firmicutes, Bacteroidota, Fusobacteriota, Proteobacteria, Actinobacteriota, Campilobacterota, and others, while the most predominant genera were *Anaerococcus*, *Fusobacterium*, *Bacteroides*, *Escherichia-Shigella*, *Finegoldia*, *Porphyromonas*, *Collinsella*, *Lactobacillus*, *Ruminococcus_gnavus_group*, *Prevotella*, and others. Different microbial communities between the Ragdoll group and the Felinae group were observed, and the compared results demonstrated that the relative abundances of beneficial microbes (such as *Lactobacillus*, *Enterococcus*, *Streptococcus*, *Blautia*, *Roseburia*, and so on) in the Ragdoll group were much higher than in the Felinae group. The co-occurrence network revealed that the number of nodes and links in the Felinae group was significantly higher than the Ragdoll group, which meant that the network of the Felinae group was larger and more complex than that of the Ragdoll group. PICRUSt function analyses indicated that the differences in microbial genes might influence the energy metabolism and immune functions of the host. In all, our data demonstrated that the richness and diversity of beneficial microbes in the Ragdoll group were much higher than the Felinae group. Therefore, it is possible to isolate and identify more candidate probiotics in the gut microbiota of growing Ragdoll cats.

## 1. Introduction

As a species of small Carnivora mammals, the domestic cat (*Felis catus*) emerged during the Miocene, about 10–11 million years ago [1]. During the long-term evolutionary process, the morphological and genetic portraits of domestic cats changed significantly from their wild ancestors [2]. Moreover, the behaviors and personalities of domestic cats are also quite different from their wild ancestors [3]. Today, domestic cats have adapted to the domesticated environment and have become popular pets all over the world. In fact, most domestic cats can not only bring mental happiness to their owners but can also provide health benefits through microbial communication. The hygiene hypothesis has demonstrated that early-life exposure to household furry pets can help the owners to establish a microbial balance and maintain immune system function, which could reduce the chances of obesity, diabetes, and allergic diseases [4,5].

The acquisition and formation of the infant gut microbiome can be influenced by both endogenous and exogenous factors, including delivery mode, antibiotic usage, bottle-feeding, pet exposure, and other factors [6,7,8]. Accumulating research has revealed that children who grew up in pet-owning families have lower chances of developing asthma than their peers who grew up in families without cats and dogs. A possible reason might be the fact that frequent exposure to household pets enhances the gut microbial diversities of their young owners [9]. Generally, the development of a host’s immune system is intimately related to the gut microbiome. The maturation of commensal microbes can decrease the rates of allergic diseases by enhancing the host’s immune function [10]. In particular, infants who grow up with proper exposure to cats, dogs, or farm animals have more chances to contact diverse microorganisms on pet fur and muddy paws; therefore, furry household pets play a critical role in transferring microbes from one person to another person.

Recently, the rapid development of high-throughput sequencing technology and other molecular analyzing approaches have deciphered the bacterial communities in feline commensal microbiome. The predominant microbes in the feline gut microbiota were found to have a certain similarity to those in the human gut microbiota [11]. Because the various kinds of metabolites produced by the gut microbiome can influence the health states of the intestine tract, the complicated relationships between the intestinal microbiota and symbiotic microbes in other organs (such as the brain, mouth, nose, and lung) are worthy of further study [12]. Well-balanced gut microbiota can provide immune protections for the host; however, a disturbed gut microbiome can cause feline inflammatory bowel disease, asthma, and other respiratory diseases [13].

The nutrient contents of fiber, starch, and protein can influence the compositions of feline gut microbiota; therefore, changes in diet structure can induce rapid microbiome shifts and metabolism profile alterations [14]. In fact, the modulations of the gut microbial composition by diet and the aging process can affect the body’s health and insulin sensitivity and can eventually induce the incidence of chronic diseases (such as Type 2 diabetes) [15]. The recent trends of “natural” and “species-appropriate” diets in human nutrition have influenced the feeding fashions of domestic cats. More utilization of protein and fat in the feline diet can increase the relative abundance of *Clostridium* and bring additional benefits by producing butyrate and other short-chain fatty acids [16]. The proper ratio of protein to carbohydrates in cat food plays an important role in maintaining mucin glycan availability and branched-chain fatty acids metabolism. Milk oligosaccharides can promote the growth of beneficial bacteria and maintain intestinal homeostasis by enhancing the production of short-chain fatty acids [17,18]. Moreover, prebiotics and probiotics can also confer multiple physiological benefits for the cats by inhibiting the growth of enteric pathogens [19,20,21].

In this study, the diversity and richness of gut microbiota in Ragdoll cats and Felinae cats were analyzed by 16S rRNA gene sequencing techniques, and the microbial communities of the feline gut microbiota were investigated and compared.

## 2. Materials and Methods

### 2.1. Animals and Diets

**Figure 1:**
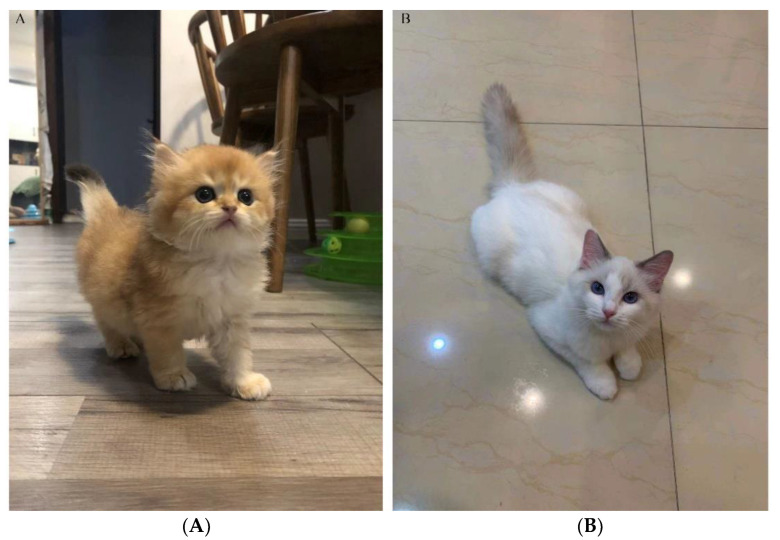
The two breeds of pedigree cats in this study. (**A**) Felinae cats and (**B**) Ragdoll cats.

A total of 13 healthy young cats ranging from 8 weeks old to 12 weeks old were enrolled in the current study (Figure 1). The growing domestic cats were separated into two groups according to their breeds: the Ragdoll cat group (2 male and 2 female) and the Felinae cat group (4 male and 5 female). At the initial enrollment, all the cats were observed for one week for any developing gastrointestinal diseases; all the cats were normally vaccinated and did not receive any antibiotic treatment. Additionally, no probiotics were provided to any of these cats prior to the study [22].

In this study, all the enrolled growing cats were housed in the same foster home and kept on the same diet. The nutrient compositions of the diet provided by the manufacturer (Lichong, Shanghai, China) are shown in Table 1, with a feline vitamin and mineral premix added. The main ingredients in the diet were composed of poultry byproducts, maize gluten, soybean, brewers rice, ground yellow maize, ground wheat, and animal fat [23]. The diet met the nutritional recommendations for all life stages of cats. At the end of the study, all the cats were eventually adopted by private homes. The protocol (number: SHVRI-fe-20210618) was approved by the experimental animal administration and ethics committee of the Shanghai Veterinary Research Institute, Chinese Academy of Agricultural Sciences.

### 2.2. Sample Collection

To collect the fecal swab, a sterile cotton tip swab was gently inserted into the rectal tract at a minimum of 2 cm, avoiding contact with the perianal area. Promptly after collection, the fecal swabs were added into 5 mL of sterile saline solution and placed on ice for transport to the laboratory [24]. All the collected fecal samples were centrifuged at 3000 rpm, the supernatants were discarded, and the bacterial pellets were stored at −80 °C for further use.

### 2.3. Fecal DNA Extraction

The microbial genome DNA was extracted using the methods previously described [25]. Briefly, an InviMag^®^ Stool DNA kit (Invitek, Berlin, Germany) was used with agitation in a mini-bead beater (Biospec Products, Bartlesville, OK, USA). The bacterial pellets were added to a 2 mL screw-cap tube containing 1 mL of lysis buffer and 0.3 g Zirconia beads (0.1 mm, Biospec Products, Bartlesville, OK, USA). After sufficient homogenization for approximately 5 min, bead beating was performed for 1 min. Then, the fecal DNA was extracted according to the manufacturer’s instructions.

### 2.4. PCR Amplification and Next-Generation Sequencing

The extracted DNA was used as the template to amplify the V3-V4 region of 16S rRNA genes, and the bar-coded primers 338F (5′-ACTCCTACGGGAGGCAGCA-3′) and 806R (5′-GGACTACHVGGGTWTCTAAT-3′) were used for the PCR reactions. The amplified PCR products were purified by a QIAquick Gel Extraction Kit (QIAGEN, Hilden, Germany, cat# 28706); then, the purified PCR products were used to construct an amplicon library [26]. Subsequently, the purified amplicons were paired-end sequenced on an Illumina MiSeq PE300 platform/NovaSeq PE250 platform (Illumina, San Diego, CA, USA) by Majorbio Bio-Pharm Technology Co. Ltd. (Shanghai, China). The raw reads were deposited into the NCBI Sequence Read Archive (SRA) database (Accession Number: PRJNA849375).

### 2.5. Bioinformatics Analysis

The raw 16S rRNA gene sequencing reads were demultiplexed, quality-filtered by fastp version 0.20.0, and merged by FLASH version 1.2.7 (http://www.cbcb.umd.edu/software/flash, accessed on 3 September 2022, version 1.2.7). All the retained sequences were processed using the QIIME 2 (Quantitative Insights into Microbial Ecology) package [27]. The operational taxonomic units (OTUs) with 97% similarity were clustered using UPARSE version 7.1 (http://drive5.com/uparse/, accessed on 3 September 2022), and the taxonomy of each OTU representative sequence was analyzed by RDP Classifier version 2.2 (http://rdp.cme.msu.edu/, accessed on 3 September 2022) against the 16S rRNA database (Silva v138). The bioinformatics analyses of all the sequenced samples referred to previous studies [28,29,30].

## 3. Results

### 3.1. OTUs Clustering

All the samples of the Ragdoll cat group and the Felinae cat group were sequenced using the 16S rRNA gene, and the high-quality reads were generated from the merged clean sequences after OTU (97% sequence similarity) clustering. A total of 658,037 high-quality sequences were obtained with an average length of 413 bp (Table 2). Taxonomic analysis of the 600 qualified OTUs revealed 15 bacteria phyla, 25 classes, 65 orders, 112 families, 250 genera, and 411 species.

### 3.2. Microbial Diversity Analysis of the Feline Gut Microbiota

**Figure 2:**
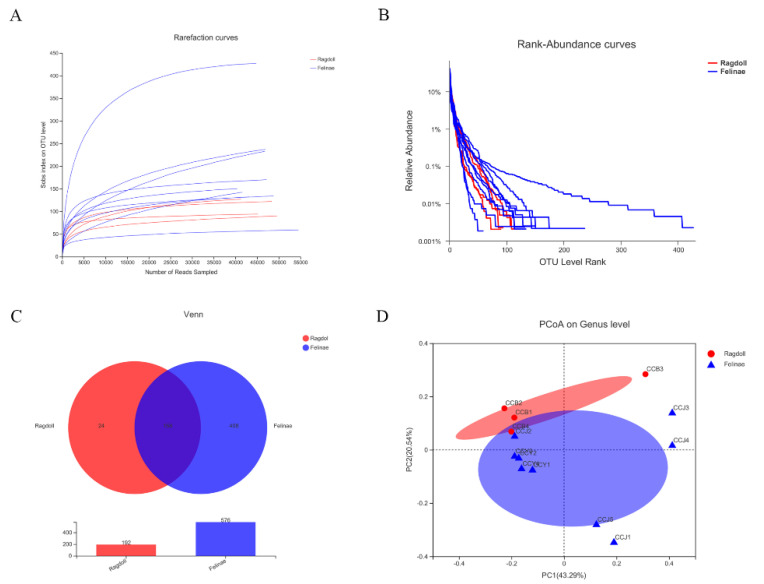
Diversities of the feline gut microbiota. The rarefaction curves (**A**) revealed that the current sequence coverage was adequate for microbial community analysis. The rank–abundance curves (**B**) indicated that the bacterial richness of the Felinae cat group was much higher than that of the Ragdoll cat group. The Venn diagram (**C**) displayed the shared and unique OTUs between the two groups. The plot of principal coordinate analysis (PcoA) (**D**) revealed the beta diversities of the feline gut microbial communities.

As shown in Figure 2A, the rarefaction curves indicated by the Sobs estimators revealed that the current sequence coverage was adequate for the microbial community analysis. The rank–abundance curves indicated that the bacterial richness of the Felinae group was much higher than that of the Ragdoll group (Figure 2B). The Venn diagrams demonstrated that there were 168 shared OTUs between the two groups, while there were 24 unique OTUs in the Ragdoll group and 408 unique OTUs in the Felinae group (Figure 2C). The number of OTUs indicated that the diversity of bacterial communities in the Felinae group was much higher than the Ragdoll group. The principal coordinate analysis (PCoA) revealed that the beta diversities of the two groups were quite different, and the microbial communities of the two groups were segregated into different clusters, respectively (Figure 2D).

### 3.3. Microbial Compositions of the Feline Gut Microbiota

The RDP classifier was used to assign the bacterial taxonomic communities of the feline gut microbiota.

**Figure 3:**
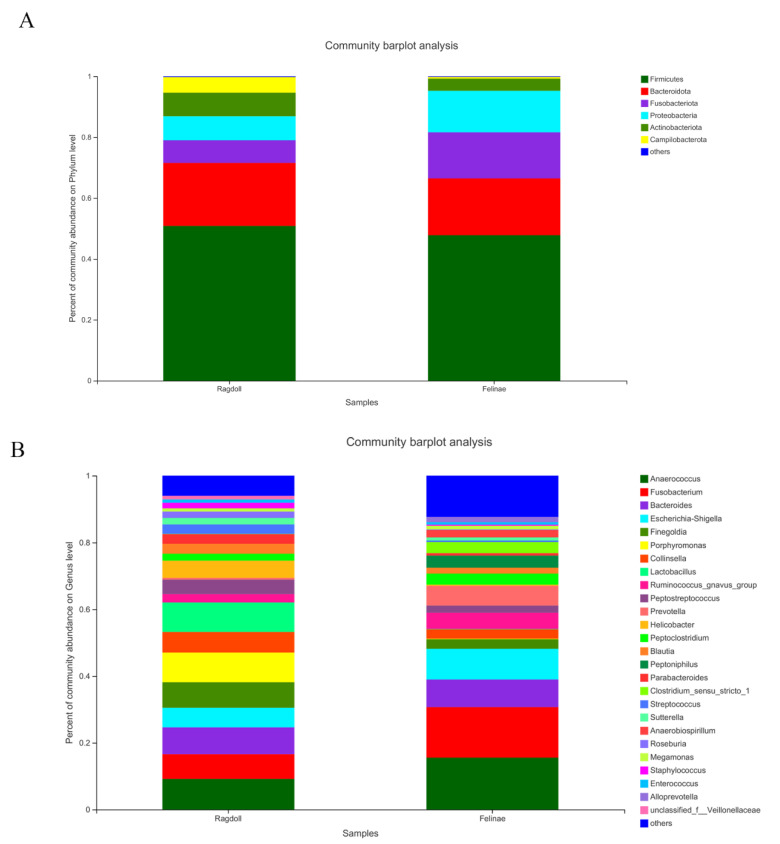
The compositions of the feline gut microbiota at the phylum (**A**) and genus (**B**) levels.

As shown in Figure 3A, the most predominant microbial communities at the phylum level were Firmicutes (49.31%), Bacteroidota (19.69%), Fusobacteriota (11.28%), Proteobacteria (10.77%), Actinobacteriota (5.87%), and Campilobacterota (2.85%). As shown in Figure 3B, the most predominant microbial communities at the genus level were *Anaerococcus* (12.37%), *Fusobacterium* (11.28%), *Bacteroides* (8.15%), *Escherichia-Shigella* (7.54%), *Finegoldia* (5.26%), *Porphyromonas* (4.53%), *Collinsella* (4.49%), *Lactobacillus* (4.48%), *Ruminococcus_gnavus_group* (3.69%), *Prevotella* (3.23%), *Helicobacter* (2.74%), *Peptoclostridium* (2.69%), *Blautia* (2.39%), *Peptoniphilus* (1.82%), *Parabacteroides* (1.80%), *Clostridium_sensu_stricto_1* (1.70%), *Streptococcus* (1.62%), *Sutterella* (1.44%), *Anaerobiospirillum* (1.08%), *Roseburia* (1.04%), *Megamonas* (1.02%), *Staphylococcus* (1.01%), *Enterococcus* (0.87%), and *Alloprevotella* (0.78%). The relative abundances of the most predominant taxa of the Ragdoll cat group and the Felinae cat group are shown in Table 3, respectively.

### 3.4. Comparison of Feline Gut Microbial Communities

**Figure 4:**
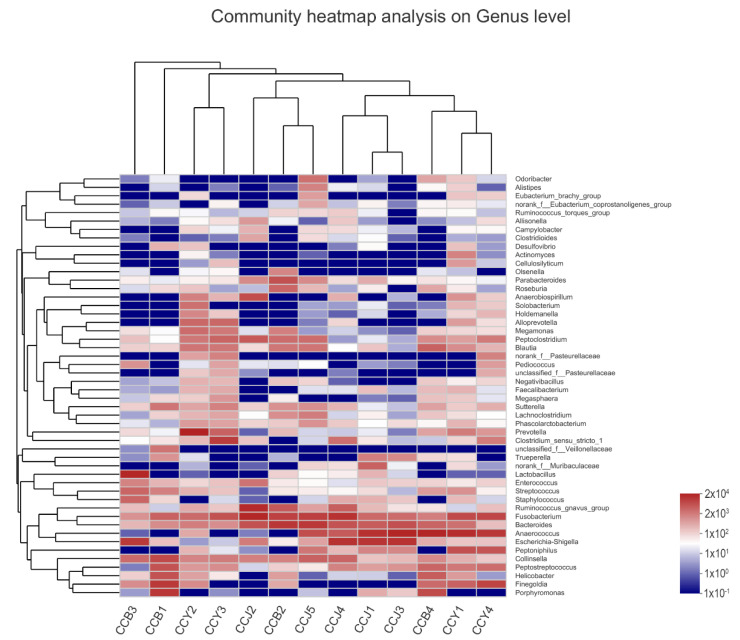
Heatmap of the hierarchy cluster results for the abundance of genus. The color of the spots corresponds to the normalized and log-transformed relative abundance, and the genus names are shown on the right.

To observe the different microbial communities between the Ragdoll group and the Felinae group, a heatmap was generated to display the top 50 genera in each sample. As shown in Figure 4, the microbial compositions between the two groups at the genus level obviously differed. The relative abundances of certain beneficial microbes in the Ragdoll group were much higher than the Felinae group, such as *Lactobacillus* (8.84% vs. 0.12%), *Enterococcus* (1.00% vs. 0.74%), *Streptococcus* (2.80% vs. 0.44%), *Blautia* (8.84% vs. 0.12%), *Roseburia* (1.92% vs. 0.17%), and so on. However, the relative abundances of some opportunistic pathogens in the Ragdoll group were much lower than the Felinae group, such as *Escherichia-Shigella* (5.86% vs. 9.21%), *Fusobacterium* (7.41% vs. 15.15%), and so on. According to the observed microbial communities, the gut microbiota of the Ragdoll group were much healthier than that of the Felinae group.

**Figure 5:**
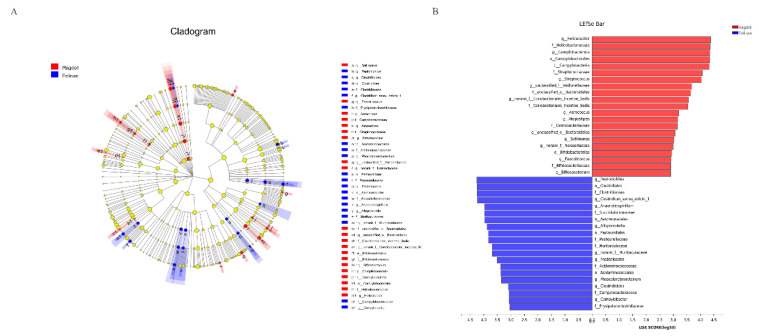
The linear discriminant analysis effect size (LEfSe) reveals the most featured bacterial taxa in the feline gut microbiota. The cladogram represents the discriminative features of the taxa between the two groups (**A**). The LDA coupled with effect size measurements identified the most differentially abundant taxa between the two groups (**B**).

The linear discriminant analysis effect size (LEfSe) was performed to identify the specific bacterial taxa between the Ragdoll group and the Felinae group (Figure 5). The largest differences in the feline gut microbiota at different taxon levels between the two groups were compared and displayed by a cladogram (Figure 5A). The microbiological markers at different phylogenetic levels of the fecal microbiota were also analyzed using the Kruskal–Wallis rank sum test, and the predominant bacteria with a linear discriminant analysis (LDA) score > 2 are shown in Figure 5B. Compared through the LEfSe analysis, the relative abundances of *Helicobacter*, *Streptococcus*, *Aerococcus*, *Atopostipes*, *Sellimonas*, *Faecalicoccus*, and *Bifidobacterium* in the Ragdoll group were significantly higher than in the Felinae group. However, the relative abundances of *Peptoniphilus*, *Clostridium_sensu_stricto_1*, *Anaerobiospirillum*, *Alloprevotella*, *Frederiksenia*, *Phascolarctobacterium*, *Clostridioides*, and *Campylobacter* in the Felinae group were significantly higher than in the Ragdoll group.

### 3.5. Co-Occurrence Network of the Feline Gut Microbiota

**Figure 6:**
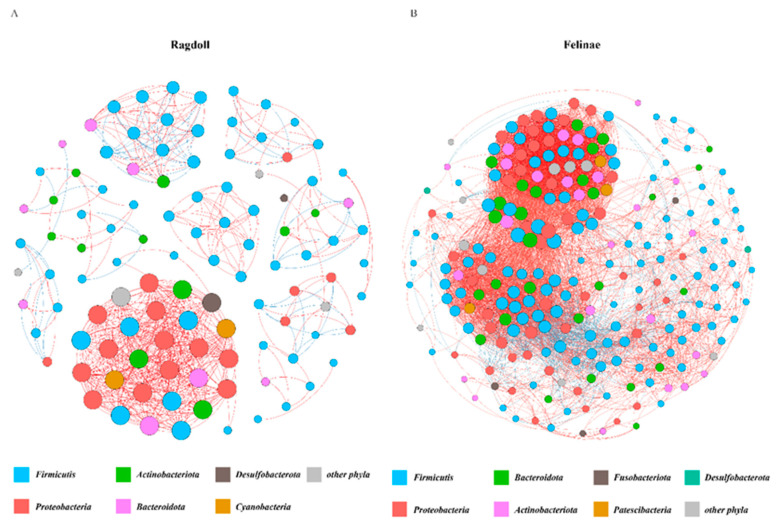
The co-occurrence patterns of feline gut microbiota. The size of each node is proportional to the number of degrees. Major phylum (with nodes > 5) are randomly colored. Positive links between nodes are red, and negative links are blue.

The molecular ecological network method based on random matrix theory (RMT) was used to construct the networks of the feline gut microbiota. The nodes that were detected in half or more of the total sample were retained for the network analysis, and the Spearman rank correlation was used to establish a co-occurrence network among the feline gut microbial communities. The intra-module connectivity value and inter-module connectivity value for each node were used to identify the keystone species in the network. The OTUs in the network mainly belonged to Firmicutes, Bacteroidota, Fusobacteriota, Patescibacteria, Proteobacteria, Actinobacteriota, and Desulfobacterota (Figure 6).

When the networks for the Ragdoll group and the Felinae group were compared, the network for the Felinae group was larger and more complex than the network for the Ragdoll group. The number of nodes in the Felinae group was twice more than that in the Ragdoll group. In addition, the number of links between nodes and the average connectivity in the Felinae group were significantly higher than in the Ragdoll group (Table 4).

### 3.6. PICRUSt Functional Prediction

**Figure 7:**
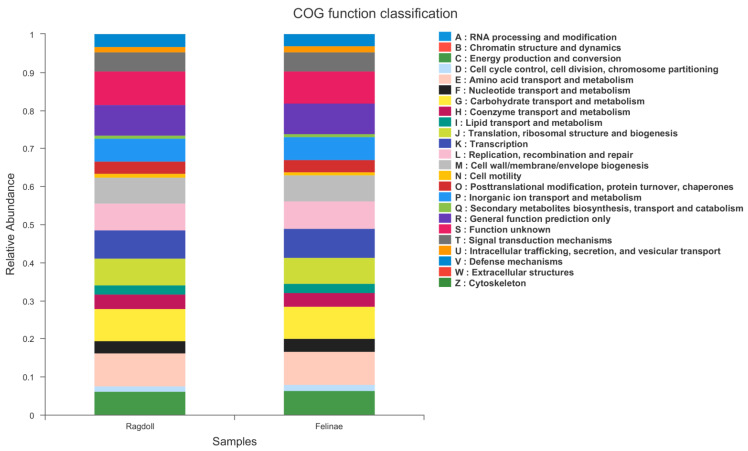
PICRUSt functional prediction was performed using EggNOG database; more than 22 pathways related to feline health were identified.

A total of 22 pathways related to feline health and metabolism were predicted using the PICRUSt in EggNOG (evolutionary genealogy of genes: Non-supervised Orthologous Groups). As shown in Figure 7, the following predicted functions were higher in the Felinae group than in the Ragdoll group: RNA processing and modification; chromatin structure and dynamics; energy production and conversion; amino acid transport and metabolism; lipid transport and metabolism; replication, recombination and repair; secondary metabolites biosynthesis, transport and catabolism; intracellular trafficking, secretion, and vesicular transport; extracellular structures; and cytoskeleton. However, the following types of microbial genes were lower in the Felinae group than in the Ragdoll group: those related to cell cycle control, cell division, chromosome partitioning; nucleotide transport and metabolism; carbohydrate transport and metabolism; coenzyme transport and metabolism; translation, ribosomal structure and biogenesis; cell motility; posttranslational modification, protein turnover, and chaperones; inorganic ion transport and metabolism; signal transduction mechanisms; and defense mechanisms. Therefore, the differences in microbial communities and microbial genes might significantly influence the energy metabolism and immune functions of the host.

## 4. Discussion

Domesticated cats have become very popular companion animals and play important roles in an owner’s life. At first, humans probably domesticated cats because they had the ability to catch rodents and could protect crops [2]. In modern life, domesticated cats mainly play a role in the emotional care and mental happiness of their owners [3,31]. In fact, frequent contact with domesticated cats can alter the owner’s microbial communities and reduce the risk of obesity and allergic diseases [9,32]. Therefore, investigations on the microbial communities in the feline gut might provide critical knowledge on the intimate associations between feline gut microbiota and human gut microbiota.

Domesticated cats’ appearances and behaviors changed significantly over time due to artificial selection [33]. In fact, the host genes play a critical role in shaping the structures of the gut microbiome [34,35]. Therefore, the distinctive morphological differences between different breeds are intimately related to cats’ genetic portraits and might also have certain relations with cats’ unique gut microbiota. In this research, the microbial diversities and communities of Ragdoll cats and Felinae cats were analyzed and compared. By quality-filtering and OTU clustering on the sequenced data, 658,037 high quality sequences belonging to 15 bacteria phyla, 25 classes, 65 orders, 112 families, 250 genera, and 411 species were obtained (Table 2). As shown in Figure 2, the bacterial richness and diversity of the Felinae cats were much higher than those of the Ragdoll cats, and the principal coordinate analysis (PCoA) revealed that the beta diversities between the two groups were obviously different.

The relative abundances of the predominant taxa in the Ragdoll group and the Felinae group were quite different (shown in Table 3). The taxonomic analyses demonstrated that the gut microbes in the two groups of feline gut microbiota were obviously different. At the phylum level, the most predominant phyla in the Ragdoll and Felinae groups were mainly composed of Firmicutes (50.83% vs. 47.80%), Bacteroidota (20.73% vs. 18.66%), Fusobacteriota (7.41% vs. 15.16%), Proteobacteria (7.91% vs. 13.63%), Actinobacteriota (7.74% vs. 4.00%), and Campilobacterota (5.14% vs. 0.55%), respectively (Figure 3A). When compared at the genus level, the most predominant genera in the Ragdoll and Felinae groups were mainly composed of *Anaerococcus* (9.19% vs. 15.55%), *Fusobacterium* (7.41% vs. 15.15%), *Bacteroides* (8.04% vs. 8.26%), *Escherichia-Shigella* (5.86% vs. 9.21%), *Finegoldia* (7.66% vs. 2.86%), *Porphyromonas* (8.90% vs. 0.15%), *Collinsella* (4.49%), *Lactobacillus* (8.84%vs. 0.12%), *Ruminococcus_gnavus_group* (2.51% vs. 4.88%), *Peptostreptococcus* (4.33% vs. 2.14%), *Prevotella* (0.54% vs. 5.93%), *Helicobacter* (5.14% vs. 0.33), *Peptoclostridium* (2.07% vs. 3.30%), *Blautia* (2.99% vs. 1,79%), *Peptoniphilus* (0.00% vs. 3.63%), *Parabacteroides* (2.87% vs. 0.72%), *Clostridium_sensu_stricto_1* (0.88% vs.3.33%), *Streptococcus* (2.80% vs. 0.44%), *Sutterella* (1.94% vs. 0.95%), *Anaerobiospirillum* (0.00% vs. 2.16%), *Roseburia* (1.92% vs. 0.17%), *Megamonas* (0.95% vs. 1.09%), *Staphylococcus* (1.68% vs. 0.33%), *Enterococcus* (1.00% vs. 0.74%), and *Alloprevotella* (0.00% vs. 1.57%), respectively (Figure 3B). As shown in Figure 4, the relative abundances of *Lactobacillus*, *Enterococcus*, *Streptococcus*, *Blautia*, *Roseburia*, and other beneficial microbes in the Ragdoll group were much higher than in the Felinae group. However, the relative abundances of *Escherichia-Shigella*, *Fusobacterium*, and other opportunistic pathogens in the Ragdoll group were much lower than in the Felinae group. Generally, the microbial compositions of the Ragdoll group were much healthier than those of the Felinae group (Figure 5). Through regulating the intestinal immune function and protecting the intestinal barrier integrity, the gut microbiota might provide additional health benefits for the host.

In the early life, the formation and maturation of the intestinal microbiota have important impacts on the development of the host’s immune system and might influence the host’s health for their whole life [36]. The diversity and richness of the beneficial microbes in the intestinal tract of young healthy cats are higher than those in older cats. Therefore, it is quite possible to isolate and identify candidate probiotics in the gut microbial communities of growing cats [37]. Simultaneously, the beneficial bacteria strains could help to maintain physiological function by producing beneficial metabolites, such as acetate, propionate, butyrate, lactic acids, antibacterial peptides, and other metabolic compounds. The antimicrobials found in the feline commensal bacterium were proved capable of fighting against drug-resistant bacteria (such as methicillin-resistant Staphylococcus pseudintermedius, MRSP)-induced infections [37].

The addition of potential probiotics in the diets of young cats could help to improve their growth development, promote the mucosal barrier maturation, and avoid the invasion of enteric pathogens [38,39]. Therefore, the feline gut microbiome could be explored as a novel therapeutic target for treating gastrointestinal tract diseases. Moreover, alterations in gut microbiota might also influence the commensal microbiome in other organs. Proper usage of probiotics could also slow inflammatory conditions and change curly and straight hair [40]. In all, manipulations of the gut microbiome might also be used to treat cats’ respiratory tract diseases and skin infections.

## 5. Conclusions

In this study, we analyzed and compared the structure of gut microbiota in Ragdoll cats and Felinae cats. Although the diversity and richness of the gut microbiota in the Felinae cats were much higher than the Ragdoll cats, the relative abundances of beneficial microbes (such as *Lactobacillus*, *Enterococcus*, *Streptococcus*, *Blautia*, *Roseburia*, and so on) in the Ragdoll group were much higher than in the Felinae group. Taken together, it is possible to isolate and identify more candidate probiotics in the gut microbiota of growing Ragdoll cats.

## Figures and Tables

**Table 1:** Primary ingredients of the utilized diets.

Ingredients	Nutrient Composition
Crude protein	40%
Crude fat	20%
Crude fiber	9.0%
Crude ash	10.0%
Calcium	1.0%
Phosphorus	0.8%
Taurine	0.2%
Chloride	0.3%
Moisture	10%
Energy	14,585 kJ/kg

**Table 2:** Summary of sequencing data.

Amplified Region	Samples	Sequences	Bases (bp)	Average Length
338F_806R	13	658,037	271,867,357	413

**Table 3:** The predominant taxonomic profiles of feline gut microbiota.

Taxonomic Level	Taxa	Felinae (%)	Ragdoll (%)
Phylum	Firmicutes	47.80	50.83
	Bacteroidota	18.66	20.73
	Fusobacteriota	15.16	7.41
	Proteobacteria	13.63	7.91
	Actinobacteriota	4.00	7.74
	Campilobacterota	0.55	5.14
Genus	*Anaerococcus*	15.55	9.19
	*Fusobacterium*	15.15	7.41
	*Bacteroides*	8.26	8.04
	*Escherichia-Shigella*	9.21	5.86
	*Finegoldia*	2.86	7.66
	*Porphyromonas*	0.15	8.90
	*Collinsella*	2.82	6.17
	*Lactobacillus*	0.12	8.84
	*Ruminococcus_gnavus_group*	4.88	2.51
	*Peptostreptococcus*	2.14	4.33
	*Prevotella*	5.93	0.54
	*Helicobacter*	0.33	5.14
	*Peptoclostridium*	3.30	2.07
	*Blautia*	1.79	2.99
	*Peptoniphilus*	3.63	0.00
	*Parabacteroides*	0.72	2.87
	*Clostridium_sensu_stricto_1*	3.33	0.08
	*Streptococcus*	0.44	2.80
	*Sutterella*	0.95	1.94
	*Anaerobiospirillum*	2.16	0.00
	*Roseburia*	0.17	1.92
	*Megamonas*	1.09	0.95
	*Staphylococcus*	0.33	1.68
	*Enterococcus*	0.74	1.00
	*Alloprevotella*	1.57	0.00

**Table 4:** The topological properties for the co-occurrence networks.

Network Features	Ragdoll	Felinae
Number of Nodes	107	240
Number of Edges	579	4932
Average Degree	10.822	41.1
Average Path Length	1	2.455
Density	0.102	0.172
Modularity	0.605	0.393
Average Clustering Coefficient	1	0.648

## Data Availability

The raw reads were deposited into the NCBI Sequence Read Archive (SRA) database (Accession Number: PRJNA849375).
